# Anticancer Effects of Ursi Fel Extract and Its Active Compound, Ursodeoxycholic Acid, in FRO Anaplastic Thyroid Cancer Cells

**DOI:** 10.3390/molecules26175309

**Published:** 2021-09-01

**Authors:** Hyo Won Jung, Ji Hye Hwang

**Affiliations:** 1 Department of Herbology, College of Korean Medicine, Dongguk University, Dongdae-ro 123, Gyeongju 38066, Korea; tenzing2@hanmail.net; 2 Department of Acupuncture & Moxibustion Medicine, College of Korean Medicine, Gachon University, Seongnam 13120, Korea

**Keywords:** thyroid cancer, Ursi Fel, ursodeoxycholic acid, anticancer effect, apoptosis, angiogenesis

## Abstract

Anaplastic thyroid cancer (ATC) is one of the most fatal human malignancies. Ursi Fel (UF) is the bile of a brown bear that has been traditionally used for heat clearance and toxin relief in Korean and Chinese medicines. In this study, we determined the anticancer effects of a UF extract and its active compound, ursodeoxycholic acid (UDCA), in FRO human ATC cells. FRO cells were treated with UF extract and UDCA at different concentrations for various durations. Cell viability was measured using an MTT assay. Cell apoptosis was investigated by flow cytometric analysis following Annexin V and propidium iodide (PI) staining, and Hoechst staining was used to observe nuclear fragmentation. The expression of pro-apoptotic (Bax, caspase-3, cytochrome c, and PARP), anti-apoptotic (Bcl-2), and angiogenetic (TGF-β, VEGF, N-cadherin, and sirtuin-1) proteins and the phosphorylation of Akt and mechanistic target of rapamycin (mTOR) were determined by western blot analysis. Treatment with UF extract at 10, 25, and 50 μg/mL and UDCA at 25, 50, and 100 μM/mL significantly inhibited the growth of FRO cells in a dose-dependent manner. Flow cytometry and Hoechst staining revealed an increase in the apoptosis of FRO cells mediated by UF extract and UDCA in a dose-dependent manner. UF extract (25 and 50 μg) and UDCA (50 and 100 μM) significantly increased the expression of Bax, caspase-3, cytochrome c, and PARP and inhibited the expression of Bcl-2, TGF-β, VEGF, N-cadherin, and sirtuin-1 in FRO cells. Furthermore, UF extract and UDCA treatment stimulated Akt phosphorylation and inhibited mTOR phosphorylation in these cells. These results indicate that UF extract and UDCA exert anticancer properties in FRO cells by inducing apoptosis and inhibiting angiogenesis via regulating the Akt/mTOR signaling pathway.

## 1. Introduction

The incidence of thyroid cancer, one of the most common endocrine tumors, is increasing worldwide. Malignant thyroid cancer can be classified into four main types comprising papillary, follicular, medullary, and anaplastic thyroid cancer (ATC) [1,2]. ATC is one of the most lethal malignancies owing to poor differentiation and invasion into the surrounding tissues, such as the trachea [3]. The current clinical treatment regimens for ATC, including surgery and/or radiotherapy or chemotherapy, are associated with low recovery and high relapse rates, which are indicative of their ineffectiveness [4,5]. Therefore, natural sources have been exploited to discover new anticancer treatments [6].

The bile of *Ursus arctos* (a brown bear’s bile, Ursi Fel; UF) is a representative heat-clearing Korean medicine that is considered cold in character and bitter in flavor. It is associated with channel tropisms in the liver, gallbladder, and heart; thus, it may be effective in clearing heat, relieving toxins, alleviating convulsions, clearing away liver fire, and improving eyesight [7,8,9]. UF is commonly used to treat fever, inflammation, and swelling, as well as to facilitate detoxification and ameliorate pain in traditional clinics [7,8]. In addition, UF is helpful for treating hepatobiliary diseases and other liver diseases, including viral hepatitis, liver fibrosis, and liver cancer [8,9].

Among the bile acids, the main ingredients of UF reported to possess anti-neoplastic and anti-carcinogenic properties [10], ursodeoxycholic acid (UDCA), is used to treat liver diseases. UDCA is also believed to be associated with the induction of apoptosis and cell cycle arrest and suppression of the oncogenic factors, Ras and cyclooxygenase (COX)-2, in HepG2 hepatocellular carcinoma [11] and other cancer types such as gastric cancer [12] and prostate cancer [13]. In addition, UDCA is effective in inducing apoptosis following irradiation of photosensitized murine leukemia L1210 and hepatoma 1c1c7 cells [14]).

However, the anticancer mechanism of UDCA is not fully understood. Here, we investigated the anticancer effects of a UF extract and its active compound, UDCA, on the human ATC cell line, FRO, to elucidate the underlying mechanism of action. To the best of our knowledge, this is the first study to demonstrate the apoptotic and angiogenic effects of UF extract and UDCA in ATC.

## 2. Materials and Methods

### 2.1. Materials

Ursodeoxycholic acid (UDCA), docetaxel, and thiazolyl blue tetrazolium bromide (MTT) reagent were purchased from Sigma-Aldrich (St. Louis, MO, USA). Anti-Bax (sc-526), anti-B cell lymphoma-2 (Bcl-2; sc-7382), anti-caspase-3 (sc-7148), and anti-transforming growth factor (TGF)-β (sc-146) antibodies (Abs) were procured from Santa Cruz Biotechnology (Santa Cruz, CA, USA). Anti-cleaved caspase-3 (9661S), anti-poly(ADP-ribose) polymerase (PARP; #9541), anti-protein kinase B (Akt; #9272), anti-phospho-Akt (#9271), anti-mechanistic target of rapamycin (mTOR; 2972S), anti-phospho-mTOR (5536S), and anti-N-cadherin (#4061) Abs were supplied by Cell Signaling Technologies (Boston, MA, USA). Anti-vascular endothelial growth factor (VEGF) Ab (sc-146) was procured from Novus Biologicals (E. Briarwood Avenue, CO, USA), and anti-sirtuin-1 (Sirt-1) Ab (bc-0921R) was obtained from Bioss Antibodies Inc. (Woburn, MA, USA). The antibody specific for β-actin was obtained from Sigma-Aldrich Co. (St. Louis, MO, USA).

Horseradish peroxidase (HRP)-conjugated goat anti-rabbit immunoglobulin G (IgG) and goat anti-mouse IgG secondary antibodies were obtained from Bio-Rad (Hercules, CA, USA). The radioimmunoprecipitation assay (RIPA) buffer was purchased from Thermo Scientific Co. (Rockford, IL, USA), and 1× protease inhibitor cocktail kit (tissue 2 perfect) was supplied by Quartett (Berlin, Germany). Xpert phosphatase inhibitor was purchased from GenDEPOT (Barker, TX, USA), and nitrocellulose (NC) membrane and clarity enhanced chemiluminescence (ECL) western blot substrate were procured from Bio-Rad.

### 2.2. Preparation of UF Extract

UF was purchased from an herbal material company (Jayeondameun, Yangju, Korea), and UF extract was obtained as a finished product at 1 mg/mL in water and alcohol (*v*/*v* = 1:1) from a good manufacturing practice (GMP)-compliant facility (Namsangcheon, an extramural herbal dispensary facility, Yongin, Korea).

### 2.3. Cell Lines and Cell Culture

The FRO cell line, a cancer cell line from human anaplastic thyroid carcinoma, was provided by Dr. Kim E.S., Ulsan University Hospital (Ulsan, Korea), and maintained at 37 °C in a humidified atmosphere with 5% CO_2_ in Dulbecco’s modified Eagle’s medium (DMEM; Corning Incorporated, Corning, NY, USA) supplemented with 10% fetal bovine serum (Corning Incorporated). The cells were cultured in 30 mm dishes for 24 h and treated with UF extract or UDCA at different concentrations for 24, 48, and 72 h.

### 2.4. Cell Viability Assay

Cell viability was determined using an MTT assay. First, the cells (1 × 10^4^ cells/well) were seeded into 96-well plates. After overnight incubation, the cells were treated with UF extract (0, 10, 25, 50, and 100 μg) and UDCA (0, 10, 25, 50, and 100 μM) for various time points. After each incubation period, the medium was replaced with 100 μL MTT solution (5 mg/mL), and the plates were incubated for 4 h. The reaction was terminated by removing the MTT solution and adding dimethyl sulfoxide (DMSO) to each well and incubating the plate for 15 min at room temperature. The solubilized purple formazan crystals were transferred to 96-well plates (100 μL/well), and the colorimetric reaction was evaluated by measuring the optical density at 570 nm using a microplate reader (UVM, Cambridge, UK). Finally, cell viability was calculated relative to the untreated control cells. 

### 2.5. Flow Cytometry Analysis Using Annexin V and Propidium Iodide (PI) Staining

To confirm the apoptotic effects of the test compounds, cells were stained using the Muse ^TM^ Annexin V & Death Cell Kit (Cat No. MCH100105, Millipore, Taufkirchen, Germany) according to the manufacturer’s procedure. Briefly, the cells were collected, washed with 1× phosphate-buffered saline (PBS), and resuspended in a 1× binding buffer containing Annexin V and PI. After incubation for 20 min at room temperature in the dark, fluorescence intensity values for live and apoptotic cell populations were detected by a Muse ^TM^ Cell Analyzer (Millipore). The results were analyzed using the instrument’s software.

### 2.6. Hoechst Staining

The cells were seeded on Lab-Tek chamber slides (Nalge Nunc International) and cultured overnight in an incubator at 37 °C with 5% CO_2_. The cells were treated with UF extract (0, 25, and 50 μg) and UDCA (50 and 100 μM) for 48 h, washed three times with 1× PBS, fixed with cold 4% paraformaldehyde, and incubated with Hoechst 33342 dye (Thermo Fisher Scientific) for 15 min at room temperature. The stained cells were washed three times with 1× PBS and observed under a fluorescent microscope (Leica, Wetzlar, Germany).

### 2.7. Western Blot Analysis

The cells were treated with UF extract (0, 25, and 50 μg) and UDCA (50 and 100 μM) for 48 h, harvested, and lysed for isolating the total protein using RIPA Lysis and Extraction Buffer (Thermo Fisher Scientific) containing 1× protease inhibitor cocktail (tissue 2 perfect) and Xpert phosphatase inhibitor (GenDEPOT, Barker, TX, USA). The cell debris were removed by centrifugation at 16,000× *g* for 20 min at 4 °C. Equal amounts of proteins were electrophoresed (Bio-Rad, Hercules, CA, USA) onto 8–12% polyacrylamide gels containing 10% sodium dodecyl sulfate (SDS), and protein weights were determined using a protein size marker (#161-0374, Bio-Rad, Hercules, CA, USA). An electric transfer system was used to transfer proteins from the gel onto an NC membrane. Non-specific binding was blocked with 5% skim milk in TBST buffer (5 mm Tris-HCl at pH 7.6, 136 mm sodium chloride (NaCl), and 0.1% Tween-20) for 1 h at room temperature. Thereafter, the membranes were incubated with primary antibodies overnight at 4 °C, washed three times with 1× TBST, and probed for 1 h with source-matched anti-rabbit or anti-mouse secondary mAbs in 5% skim milk at room temperature. The membranes were washed three times with 1× TBST, and signals were developed using ECL western blotting detection reagents (GE Healthcare Bio-Sciences, Pittsburgh, PA, USA) before analyzing on a ChemiDoc MP Imaging System (Bio-Rad Laboratories). The intensities of western blot bands for Bax, Bcl-2, cytochrome c, caspase-3, PARP, TGF-β, mTOR, N-cadherin, and SIRT-1 were quantified against the intensity of β-actin used as an internal control. The band intensities of Akt and mTOR were calculated from the ratio of their phosphorylated forms to their total expression.

### 2.8. High-Performance Liquid Chromatography (HPLC) Analysis

To identify the constituents of the UF extract, including UDCA, HPLC was performed. The HPLC instrument was equipped with a Waters Delta 600 (Waters, Milford, MA, USA) pump, a photodiode array (PDA) detector, and a CAPCELL PAK C18 UG80 column (SHISHEIDO Co., Ltd. Japan). Chromatographic separations were performed using a gradient solvent system comprising acetonitrile with 0.1% formic acid (B) and water (A). The gradient elution was as follows: 0% B at 0 min, 3% B at 5 min, 3% B at 15 min, 10% B at 25 min, 15% B at 35 min, 15% B at 45 min, 30% B at 55 min, 50% B at 65 min, 70% B at 75 min, and 0% B 80 min. The column eluent was monitored at 280 nm. All solvents were degassed using 0.2 μm cellulose acetate filtration. Chromatography was performed at room temperature and a flow rate of 1.0 mL/min using a 25 μL sample.

### 2.9. Statistical Analysis

We used the GraphPad Prism 5.0 (GraphPad Software, Inc., San Diego, CA, USA) for statistical analyses. All experimental data were summarized as means ± standard errors of the means of three independent experiments. The comparisons between the groups were analyzed by a Student’s *t*-test and one-way analysis of variance followed by a Tukey’s post hoc test; results with *p* < 0.05 were considered statistically significant.

## 3. Results

### 3.1. Effects of UF Extract and UDCA on Cell Growth

**Figure 1:**
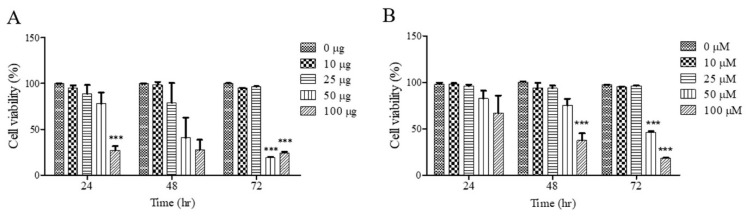
Effects of UF extract and UDCA on the viability of human FRO anaplastic thyroid cancer cells. The cells were treated with UF (**A**) or UDCA (**B**) at different concentrations for indicated time points. Cell viability was determined using the MTT assay. The data are presented as the mean ± SEM of three independent experiments. *** *p* < 0.05 vs. normal cells.

To investigate the effects of UF extract and UDCA on cell growth, FRO cells were treated with these agents at different concentrations for 24, 48, and 72 h; cell viability was measured using an MTT assay. As shown in Figure 1A, cell viability was significantly inhibited after treatment with UF extract at 100 μg/mL for 24 h (*p* < 0.05), 48 h (*p* < 0.05), and 72 h (*p* < 0.001); the half-maximal inhibitory concentration (IC_50_) was approximately 50.92 μg/mL for 48 h and 49.45 μg/mL for 72 h. As shown in Figure 1B, there was a significant dose-dependent inhibition of cell viability after treatment with UDCA at 100 μM/mL for 48 h (*p* < 0.001) and 72 h (*p* < 0.001). The (IC_50_) value was approximately 58.92 μM/mL for 48 h and 52.49 μM/mL for 72 h.

### 3.2. Effect of UF Extract and UDCA on Apoptosis

**Figure 2:**
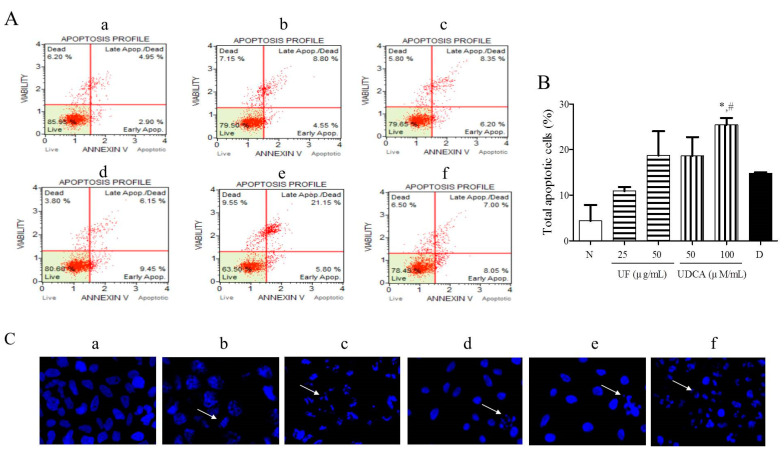
Effects of UF extract and UDCA on the apoptosis of human FRO anaplastic thyroid cancer cells. The cells were treated with UF or UDCA at indicated concentrations for 48 h: (**A**) Cell apoptosis was measured by flow cytometric analysis following Annexin V/PI staining. (**B**) Apoptotic ratios were calculated as the number of apoptotic cells per total number of cells. The data represent one of three independent experiments. *p* < 0.05 vs. normal cells (*) or docetaxel-treated cells (#). (**a**) Untreated cells; (**b**) 25 μg UF-treated cells; (**c**) 50 μg UF-treated cells; (**d**) 50 μM UDCA-treated cells; (**e**) 100 μM UDCA-treated cells; and (**f**) 2 nM docetaxel-treated cells. (**C**) Nuclear fragmentation was observed by Hoechst staining using a fluorescence microscope (×400). Arrows show apoptotic cells with nuclear fragmentation.

To investigate the effects of the UF extract and UDCA on cellular apoptosis, FRO cells were treated with different concentrations of these agents for 48 h and then stained with Annexin V/PI and Hoechst stain. Our results showed that 25 and 50 μg/mL UF extract treatment groups and 50 and 100 μM/mL UDCA treatment groups showed higher numbers of early (annexin-positive; PI-negative) and late (annexin-positive; PI-positive) apoptotic cells than the untreated control groups. The percentages of early and late apoptotic cells were 8.05% and 7.00% (Figure 2A), respectively, after treatment with 2 nM/mL docetaxel. In particular, the treatment with a high concentration of UDCA significantly (*p* < 0.05) increased the number of apoptotic FRO cells compared with untreated cells or 2 nM/mL docetaxel treatment (Figure 2B). Hoechst staining showed that the cells treated with 25 and 50 μg/mL UF or 100 μM/mL UDCA exhibited a dose-dependent increase in nuclear fragmentation (Figure 2C).

**Figure 3:**
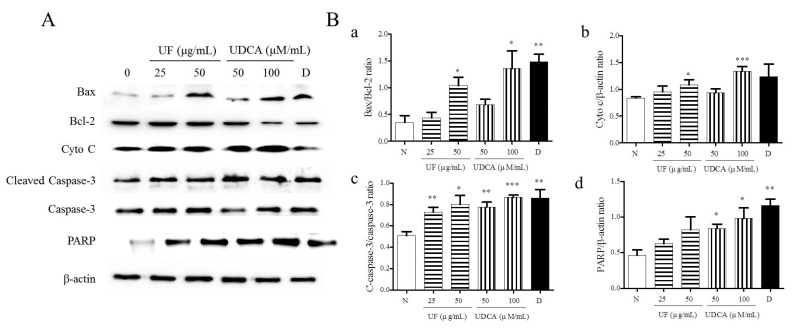
Effects of UF extract and UDCA on the expression of apoptotic proteins in human FRO anaplastic thyroid cancer cells. The cells were treated with UF extract or UDCA at indicated concentrations for 48 h. The expression of Bax, Bcl-2, cytochrome c (Cyto C), caspase-3, cleaved caspase-3 (C-caspase-3), and PARP proteins was determined by western blotting. (**A**) β-Actin was used as an internal control. The expression of Cyto C (**b**) and PARP (**d**) was calculated relative to β-actin expression. The expression of Bax was calculated relative to Bal-2 expression. (**a**) The expression of caspase-3 was calculated relative to cleaved caspase-3 expression. (**c**) The data are representative of three independent experiments. * *p* < 0.05, ** *p* < 0.01, and *** *p* < 0.001 vs. normal cells. (**B**) N, untreated cells; UF25, 25 μg/mL UF extract treatment; UF50, 50 μg/mL UF extract treatment; UDCA50, 50 μM/mL UDCA treatment; UDCA100, 100 μM/mL UDCA treatment; D, 2 nM/mL docetaxel treatment.

Next, we determined the expression of pro-apoptotic proteins, including Bax, caspase-3, cleaved caspase-3, cytochrome c, and PARP, and the anti-apoptotic protein, Bcl-2, in FRO cells using western blotting. As shown in Figure 3, the treatment with UF extract or UDCA increased the expression levels of Bax, cleaved caspase-3, cytochrome c, and PARP while inhibiting Bcl-2 expression compared with untreated cells. In particular, a significant increase in the ratio of Bax/Bcl-2 expression was observed in cells treated with 50 μg/mL UF extract (*p* < 0.05), 100 μM/mL UDCA (*p* < 0.05), and docetaxel (*p* < 0.01). An increase in cytochrome c level was observed in the cells treated with 50 μg/mL UF extract (*p* < 0.01) and 100 μM/mL UDCA (*p* < 0.001); furthermore, the ratio of cleaved caspase-3/caspase-3 expression increased after treatment with 25 (*p* < 0.01) and 50 μg/mL UF extract (*p* < 0.05) and 50 (*p* < 0.01) and 100 μM/mL UDCA (*p* < 0.001) and docetaxel (*p* < 0.01). PARP level increased in cells exposed to 50 (*p* < 0.05) and 100 μM/mL (*p* < 0.05) UDCA and docetaxel (*p* < 0.01) (Figure 3).

### 3.3. Effect of UF Extract and UDCA on the Expression of Angiogenic Regulators

**Figure 4:**
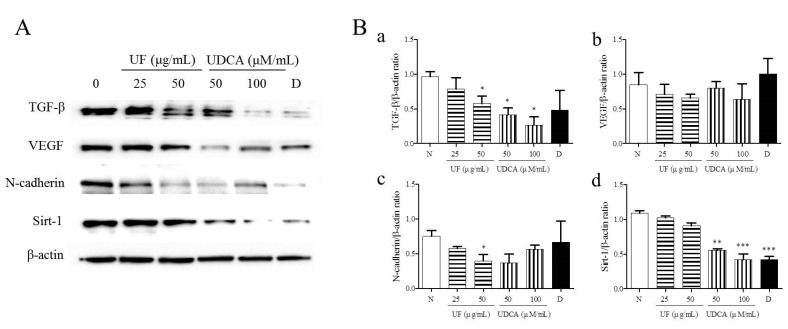
Effects of UF extract and UDCA on the expression of angiogenic proteins in human FRO anaplastic thyroid cancer cells. The cells were treated with UF or UDCA at indicated concentrations for 48 h. The expression of angiogenic proteins TGF-β (**a**), VEGF (**b**), N-cadherin (N-Cad) (**c**), and SIRT-1(d) in the cells was determined by western blotting. (**A**) β-Actin was used as an internal control. The expression of each protein was calculated relative to β-actin expression. Data are representative of three independent experiments. (**B**) * *p* < 0.05, ** *p* < 0.01, and *** *p* < 0.001 vs. normal cells. N, untreated cells; UF25, 25 μg/mL UF extract treatment; UF50, 50 μg/mL UF extract treatment; UDCA50, 50 μM/mL UDCA treatment; UDCA100, 100 μM/mL UDCA treatment; D, 2 nM/mL docetaxel treatment.

To investigate the effects of UF extract and UDCA on angiogenesis in ATC, we performed western blotting to measure the expression of angiogenic regulators, TGF-β, VEGF, N-cadherin, and SIRT-1, in FRO cells. The results showed that the treatment with UF extract and UDCA decreased the expression of TGF-β, VEGF, N-cadherin, and SIRT-1 as compared with untreated control cells (Figure 4). In particular, a significant decrease in N-cadherin expression was observed after treatment with 50 μg/mL UF extract (*p* < 0.05) and 100 μM/mL UDCA (*p* < 0.01). In addition, caspase-3 expression decreased after exposure to 50 μM/mL UDCA (*p* < 0.05). VEGF expression also decreased after treatment with UDCA at 50 (*p* < 0.05) and 100 μM/mL (*p* < 0.01) concentrations. SIRT-1 expression was downregulated after treatment with 50 (*p* < 0.01) and 100 μM/mL UDCA (*p* < 0.001) and docetaxel (*p* < 0.001) (Figure 4).

### 3.4. Effect of UF Extract and UDCA on the Akt/mTOR Pathway

**Figure 5:**
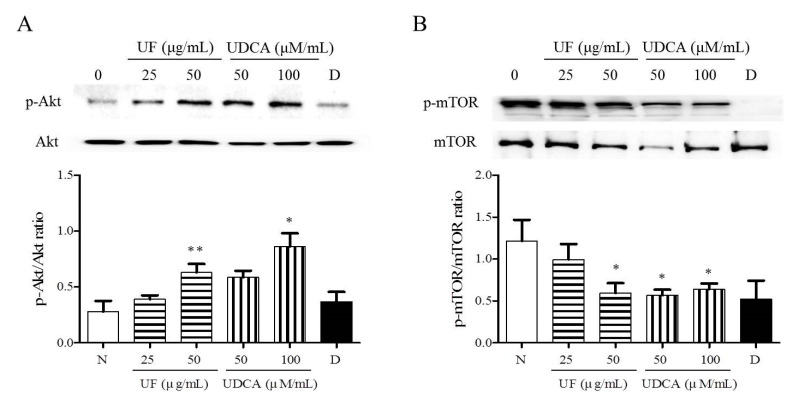
Effects of UF extract and UDCA on the phosphorylation of mTOR and Akt in human FRO anaplastic thyroid cancer cells. The cells were treated with UF extract or UDCA at indicated concentrations for 48 h. The expression of phosphorylated Akt (**A**) and mTOR (**B**) was determined by western blotting. The phosphorylation level of each protein was calculated relative to the expression of their total content. Data are representative of three independent experiments. * *p* < 0.05 and ** *p* < 0.01 vs. normal cells. N, untreated cells; UF25, 25 μg/mL UF extract treatment; UF50, 50 μg/mL UF extract treatment; UDCA50, 50 μM/mL UDCA treatment; UDCA100, 100 μM/mL UDCA treatment; D, 2 nM/mL docetaxel treatment.

To investigate the mechanism underlying the apoptotic and angiogenic effects of UF extract and UDCA in ATC, we performed western blot analysis and determined the phosphorylation levels of Akt and mTOR. These proteins are part of the main signaling pathway involved in the regulation of cell proliferation and migration [15]. Western blot results revealed significantly higher Akt phosphorylation after treatment with UF extract at 50 μg/mL (*p* < 0.01) concentrations and UDCA at 100 μM/mL (*p* < 0.05) concentrations than that in the untreated cells (Figure 5A). The mTOR phosphorylation level was significantly lower in the 50 μg/mL UF extract treatment group (*p* < 0.05), 50 (*p* < 0.01) and 100 μM/mL (*p* < 0.05) UDCA treatment groups, and docetaxel (*p* < 0.05) treatment group than in the untreated control group (Figure 5B).

### 3.5. HPLC Analysis

**Figure 6:**
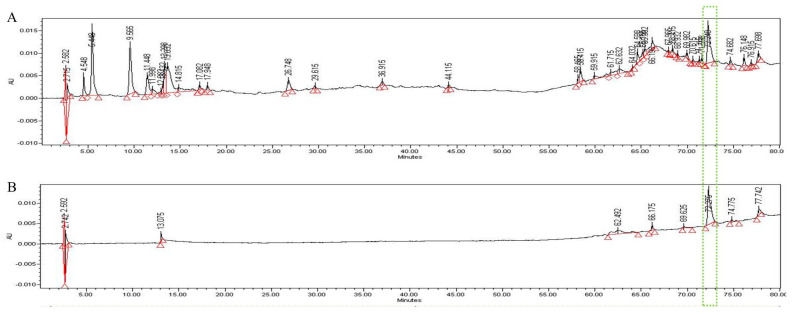
HPLC pattern for UF extract (**A**) and UDCA (**B**). The main compound in UF, UDCA, was identified based on the retention time and chromatogram pattern of an authentic standard. Peak 1 with a retention time of 72.275 min corresponds to UDCA.

In the HPLC analysis of the UF extract, UDCA was identified by comparing the retention times of authentic standards (Figure 6). We confirmed that UDCA and UF in the extract were identified at a retention time of 72.2 min after comparing with authentic standards.

## 4. Discussion

ATC is considered one of the most aggressive malignant tumors and is associated with extremely poor survival, regardless of treatment modality [16,17]. The best practices for managing ATC patients include palliative surgery to reduce tumor burden, followed by either radiotherapy and/or chemotherapy to prevent tumor progression and further metastatic growth [4]. However, despite multi-modal strategies, clinical improvement has been poor among ATC survivors, highlighting the need for new therapeutic agents [4,18]. Thus, several studies have focused on identifying natural sources that could replace synthetic drugs.

In traditional Korean and Chinese medicines, UF is used to treat fever, inflammation, and swelling and alleviate pain and facilitate detoxification. UF exhibits a wide range of pharmacological activities, including anticancer properties [8,9]. However, the widespread consumption of bear bile has made this animal an endangered species. Thus, suitable substitutes may be used soon to support the tenets of the World Animal Protection organization.

Recent reports show that bear bile is mainly composed of bile acids, amino acids, bile pigments, fats, phospholipids, and trace metals [19,20]. Bile acids exhibit anti-neoplastic and anti-carcinogenic effects against many cancer cells, including those derived from colon cancer, tamoxifen-resistant breast cancer, prostate cancer, and neuroblastoma [10]. Among the bile acids, UDCA has been recommended as one of the pure chemical substitutes for UF and was recently proposed as an effective drug for various chronic liver diseases, especially cholestatic liver disorders such as primary biliary cirrhosis [21]. The tumor inhibitory activity of UDCA is believed to be associated with the induction of apoptosis and cell cycle arrest. UDCA inhibits the expression of oncogenic factors such as Ras and COX-2 via inducing apoptosis through an extrinsic pathway in human HepG2 liver cancer cells [11] and an intrinsic pathway in DU145 prostate cancer cells [13]. Although the antitumor effects of UF and UDCA have been reported in various types of cancers, none of the studies have reported their effects in thyroid cancer. Our study revealed the anti-apoptosis and anti-angiogenic effects of UF and UDCA in FRO cells, a human ATC cell line.

Apoptosis induction in cancer cells is an important chemotherapeutic approach in which cancer cells undergo self-destruction, which minimizes the off-target effects [22,23]. The intrinsic apoptotic pathway is characterized by permeabilization of the mitochondria. Activation of the intrinsic apoptotic pathway following a stimulus leads to the opening of the mitochondrial permeability transition pores and the subsequent release of pro-apoptotic proteins such as cytochrome c and PARP. These events result in the loss of the mitochondrial membrane potential and the activation of multiple caspases such as caspase-3 [24]. The Bcl-2 family modulates the mitochondrial permeability by adjusting the balance between the expression of pro-apoptotic (Bcl-2) and anti-apoptotic (Bax) proteins [25]. In the present study, we showed that UF extract and UDCA increased apoptosis and induced nuclear fragmentation in FRO cells. Our results are consistent with those of a previous study describing the biochemical and morphological changes during the apoptosis process [26]. In addition, we found that UF extract and UDCA increased the expression of apoptotic proteins, including caspase-3, PARP, and cytochrome c, in FRO cells. These results suggest that ATC cell growth and proliferation are inhibited by UF and UDCA, resulting in the induction of apoptosis through Bax and Bcl-2 expression, mitochondrial dysfunction, caspase activation, and PARP cleavage.

The development and progression of ATC involve complex interactions between hormones and growth factors such as epithelial growth factor (EGF)-like ligands and TGF-β1, which regulate the proliferation and differentiation of thyroid cells [27]. Targeting TGF-β1 may be an effective strategy to suppress primary tumor cell proliferation in ATC [28]. VEGF, which is essential for cell growth and metastasis, is a major cytokine associated with tumor angiogenesis [29]. Most tumor types, including ATC, are known to overexpress VEGF, which is associated with neoangiogenesis. N-cadherin, an adhesion molecule, promotes tumor cell survival, migration, and invasion and is expressed in endothelial cells. N-cadherin is associated with the increased invasive potential of cancers [30]. SIRT-1, a class III histone deacetylase capable of deacetylating lysine residues in nuclear proteins, regulates vascular endothelial homeostasis by controlling angiogenesis and vascular function [31,32]. In our study, treatment with UF extract and UDCA inhibited the expression of TGF-β, VEGF, N-cadherin, and SIRT-1 in FRO cells, suggesting that these agents exert suppressive effects on tumor proliferation and invasion in ATC. However, further studies with UF extract and UDCA are warranted to understand their effects on angiogenesis and metastasis in ATC.

To clarify the mechanism of action underlying the apoptotic and anti-angiogenic effects of UF extract and UDCA, we investigated the phosphorylation of Akt and mTOR signaling proteins. Previous studies have reported alterations in the phosphoinositide 3-kinase (PI3K)/Akt/mTOR signaling pathway, which acts in a proto-oncogene manner and has attracted attention as a target for molecular biomarker-based and targeted tumor therapies [15,33]. The main negative regulator of autophagy, mTOR, is a serine/threonine-protein kinase that regulates cell growth, cell proliferation, and protein synthesis. Downregulation of Akt/PI3K expression results in the inactivation of mTOR and induction of autophagy in cancer cells [34,35,36]. Akt activation further activates the downstream mTOR signaling pathway, which is involved in gene transcription, protein translation, cell survival, and anti-apoptosis [37,38]. Therefore, the activation of the Akt/mTOR pathway plays a significant role in the migration and invasion of tumors [39,40,41,42]. In our study, mTOR phosphorylation was inhibited, and Akt phosphorylation increased in FRO cells after treatment with UF extract and UDCA. However, the different effects of UF extract and UDCA on the Akt/mTOR signaling pathway remain to be elucidated in future studies. 

Several pharmacological agents have been developed for cancer therapy. These agents specifically target and inhibit signaling pathways, alone or in combination, to provide effective treatment for therapy-resistant thyroid cancer by inhibiting tumor progression and re-differentiation [43]. Our results provide credible evidence that treatment with UF extract and UDCA could prevent cancer cell growth, proliferation, survival, and invasion by regulating the Akt/mTOR signaling pathway.

## 5. Conclusions

In conclusion, UF extract and UDCA showed anticancer effects on FRO cells via modulating the expression of apoptotic proteins, including Bax, caspase-3, PARP, and cytochrome c, and inducing nuclear fragmentation. UF extract and UDCA also inhibited the expression of angiogenic proteins such as TGF-β, VEGF, N-cadherin, and SIRT-1 by regulating the Akt/mTOR signaling pathway. Therefore, UF extract and UDCA have the potential for the treatment and prevention of ATC.

## Data Availability

Data is contained within the article. The data presented in this study are available on request from the corresponding author.

## Sample Availability

Samples of the compounds are not available from the authors.
